# Supplementary material for: The Systems Biology Research Tool: evolvable open-source software
Source: BMC Syst Biol. 2008 Jun 29;2:55. doi: 10.1186/1752-0509-2-55 (PMC2446383; doi:10.1186/1752-0509-2-55)
Supplement: Additional file 1 — SBRT Archive. An archive of the current version of the Systems Biology Research Tool. [file 1752-0509-2-55-S1.zip › sbrt-1.4.0/doc/developers_guide/api/sbrt/kernel/util/package-summary.html]

sbrt.kernel.util


|  |  |  |
| --- | --- | --- |
| **Overview** \| **Package** \| Class \| **Use** \| **Tree** \| **Deprecated** \| **Index** \| **Help** | |  |
| **PREV PACKAGE**   **NEXT PACKAGE** | **FRAMES**    **NO FRAMES**     **All Classes** |  |


---

## Package sbrt.kernel.util

Provides classes and interfaces for performing utility functions.

**See:**
  
          **Description**

| **Interface Summary** | |
| --- | --- |
| **MutableProgressMonitor** | This interface is used to represent mutable progress montiors. |
| **ProgressMonitor** | This interface is used to represent progress monitors. |

| **Class Summary** | |
| --- | --- |
| **AbstractProgressMonitor** | This abstract class is a skeleton implementation of `ProgressMonitor`. |
| **And<E>** |  |
| **BooleanRelation<E>** |  |
| **DiscreteProgressMonitor** | This class is used to monitor the progress of a process that occurs in discrete events. |
| **Or<E>** |  |
| **PercentProgressMonitor** | This class is used to monitor a process whose progress can be measured as a percentage. |
| **UnmodifiableCollection<E>** | This class is used to create an unmodifiable view of collections. |
| **UnmodifiableIterator<E>** | This class is used to create an iterator that cannot modify its underlying collection. |
| **UnmodifiableList<E>** | This class is used to create an unmodifiable view of lists. |
| **UnmodifiableMap<K,V>** | This class is used to create an unmodifiable view of maps. |
| **UnmodifiableSet<E>** | This class is used to create an unmodifiable view of sets. |
| **Util** | This class contains static methods that perform utility functions. |

## Package sbrt.kernel.util Description

Provides classes and interfaces for performing utility functions.

---


|  |  |  |
| --- | --- | --- |
| **Overview** \| **Package** \| Class \| **Use** \| **Tree** \| **Deprecated** \| **Index** \| **Help** | |  |
| **PREV PACKAGE**   **NEXT PACKAGE** | **FRAMES**    **NO FRAMES**     **All Classes** |  |


---
